# Do we need ethics in medical practice?

**DOI:** 10.4103/0970-1591.56171

**Published:** 2009

**Authors:** Nitin S. Kekre

**Affiliations:** Department of Urology, Christian Medical College, Vellore - 632 004, Tamilnadu, India. E-mail: editor@indianjurol.com

Should a medical practitioner be aware of ethics? Does knowledge of ethics make you a thoughtful and competent doctor? The society expects medical practice to be based on sound ethical principles. But then what are these principles? Who decides what is unethical? Does ethics vary among different groups, societies or countries? Why more and more medical trials are now being conducted in the third world countries? Does the poor unsuspecting population of the third world become a guinea pig for the multinational companies? To what extent can learning ethics in a medical school produce a caring physician? How many of us put ourselves or our near and dear ones in the position of the patient before making a medical decision, more so in a difficult situation.

Surgical workshop is the order of the day and has become very popular among surgeons. Are there any ethical guidelines to govern them? How often does a new surgical procedure go through proper checks and balances before being adopted widely in medical practice? This has led to see one, do one and teach one principle and has resulted in an undisciplined introduction of laparoscopy and many other similar surgical procedures in Urology and General surgery and has earned dubious distinction of the biggest unaudited free for all in the history of surgery. The terminology of surgical learning curve has gained popularity due to the mishaps, which have occurred during the learning of laparoscopy. I am sure that the aviation industry that trains pilots would be in for a rude shock if they knew how we are trained before we adopt these new procedures.

This symposium on *Medical Ethics and Legal Issues* may not necessarily discuss the above issues but if we want to salvage the reputation and integrity of our profession, we certainly need to think and draw our own “Lakshman Rekha.” Ethics should be taught in medical schools by senior medical/paramedical faculty who are either practicing (or have practiced) medicine and have the experience of making difficult medical decisions and have learnt from their mistakes.

Dr. Joseph Thomas, Professor and Head of Urology at KMC, Manipal, who has a special interest in this field and holds a degree in medical law has guest edited this symposium. I am optimistic that it would sensitize all of us toward these important issues and provide an opportunity for further deliberation. I thank Dr. Thomas and his team for their efforts.

By the time you all receive this issue you would be aware of the sudden demise of Dr. Atul Thakre. He was a brilliant urologist and the rising star in the field of Pediatric Urology. He took keen interest in the activities of the IJU. We are all deeply saddened by this loss and I feel a fitting tribute to Dr. Atul Thakre would be development of Pediatric Urology in India.

